# Research progress on Nrf2 intervention in the treatment of diabetic retinopathy

**DOI:** 10.3389/fendo.2025.1587231

**Published:** 2025-05-21

**Authors:** Yuchen Yang, Haidong Zou

**Affiliations:** ^1^ Shanghai Eye Diseases Prevention & Treatment Center/Shanghai Eye Hospital, School of Medicine, Tongji University, Shanghai, China; ^2^ Department of Ophthalmology, Shanghai General Hospital, Shanghai Jiao Tong University School of Medicine, Shanghai, China; ^3^ Department of Ophthalmology, National Clinical Research Center for Eye Diseases, Shanghai, China; ^4^ Department of Ophthalmology, Shanghai Engineering Center for Precise Diagnosis and Treatment of Eye Diseases, Shanghai, China

**Keywords:** diabetic retinopathy, Nrf2, oxidative stress, inflammation, apoptosis, angiogenesis, ferroptosis, endoplasmic reticulum stress

## Abstract

Diabetic retinopathy (DR) is a primary cause of vision loss among individuals with diabetes and represents the most prevalent microvascular complication of diabetes mellitus. Its pathophysiological mechanisms involve processes such as oxidative stress, chronic inflammation, cell apoptosis, and angiogenesis. As a core transcription factor in the antioxidant response, Nrf2 upregulates the expression of antioxidant genes through the Keap1-Nrf2-ARE pathway, hence reducing reactive oxygen species (ROS) levels in retinal cells and alleviating oxidative stress and correlated damage. By activating Nrf2, the release of pro-inflammatory cytokines is inhibited, which helps mitigate inflammation and delays DR progression through anti-apoptotic effects, suppression of angiogenesis and ferroptosis inhibition. This review highlights the Nrf2-related regulatory mechanisms and the latest research progress regarding its function in DR, offering a theoretical foundation for Nrf2-targeted DR therapies.

## 1 Introduction

Diabetic retinopathy (DR) is a prevailing microvascular complication associated with diabetes mellitus and is a primary cause of vision loss and blindness in diabetic individuals globally (1). The development of DR is intricate, involving potential risk factors including hyperglycemia, hypertension, dyslipidemia, disease continuation and pregnancy (2). It is closely associated with pathophysiological processes such as oxidative stress, inflammatory reactions, cell apoptosis, and angiogenesis. Clinically, DR is generally classified into non-proliferative stages (NPDR) and proliferative stages (PDR). The retinal changes in NPDR primarily include microaneurysms, retinal hemorrhages, hard exudates and cotton-wool spots, whereas the features of PDR comprise the formation of neovascularization in the retina, pre-retinal hemorrhages, vitreous hemorrhage, fibrous proliferation and tractional retinal detachment. Early-stage DR may be asymptomatic, with vision impairment becoming apparent only once the disease progresses to PDR or diabetic macular edema (DME). DME can occur at any stage of DR and is positively correlated with the severity of DR (3). Once DR advances to the PDR stage, the prognosis for surgical intervention is generally poor. Anti-vascular endothelial growth factor (VEGF) agents administered via intravitreal injections effectively address some diabetic microvascular changes such as retinal neovascularization and also DME; however, a significant proportion of patients show inadequate or no response to anti-VEGF therapy (3). Hence, the pursuit of developing novel therapeutic approaches for early DR intervention remains a key focus of ophthalmic research.

Oxidative stress is a fundamental mechanism underlying the onset of DR. Recent studies indicate that the nuclear factor E2-related factor 2 (Nrf2) signaling pathway, as a crucial mediator of intracellular antioxidant defense, regulates antioxidant genes like catalase (CAT) and heme oxygenase-1 (HO-1). Nrf2 is also implicated in various pathological mechanisms, including inflammation, cell apoptosis, and angiogenesis (4). Consequently, Nrf2 can be anticipated to be a promising therapeutic target for early DR intervention. This article thoroughly reviews recent advancements in related research.

## 2 The composition of the Keap1-Nrf2-ARE signaling pathway

**Figure 1:**
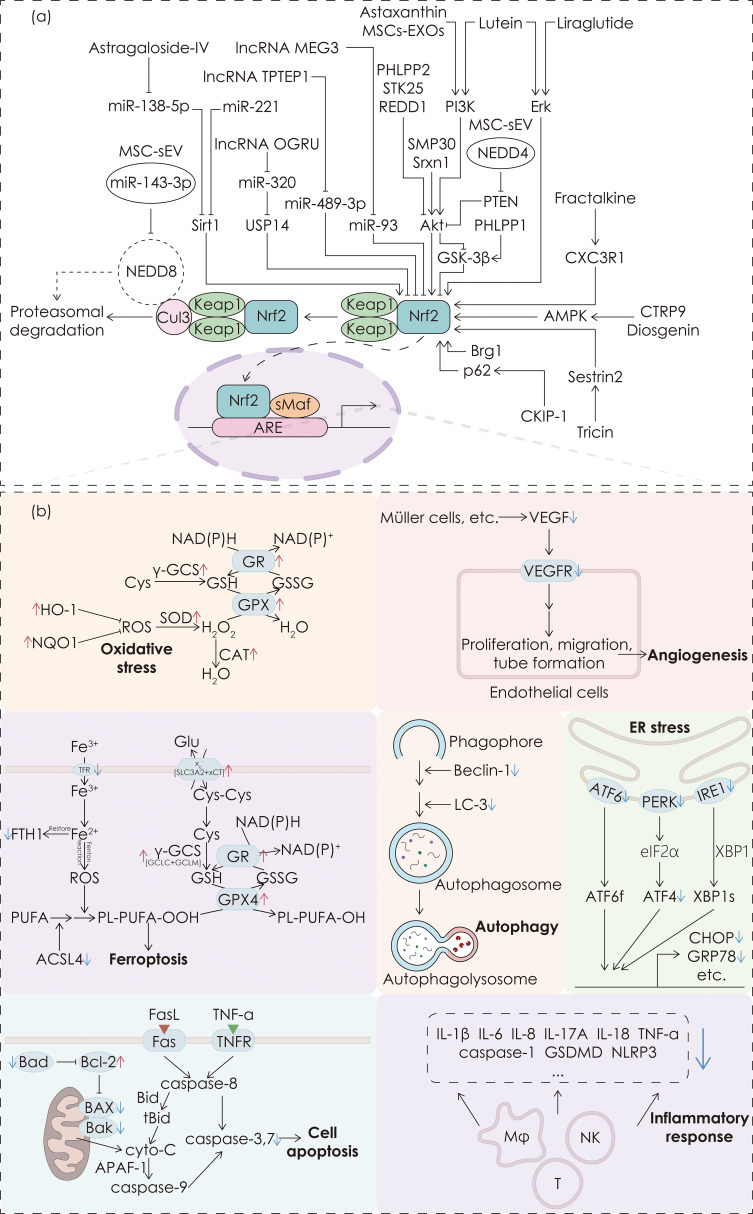
Upstream pathways of Nrf2 and downstream regulatory functions. **(a)** The mechanism of the Keap1-Nrf2-ARE pathway and interventions targeting the upstream pathways of Nrf2. **(b)** The effects induced by Nrf2. The colored blocks are arranged from top to bottom and from left to right, representing anti-oxidative stress, anti-ferroptosis, anti-apoptosis, anti-angiogenesis, anti-autophagy, anti-ER stress, and anti-inflammation effects, respectively. The upregulation or downregulation of corresponding molecules by Nrf2 is indicated by a red upward arrow or a blue downward arrow respectively next to the corresponding molecules.

Kelch-like ECH-associated protein 1 (Keap1) functions as a substrate adaptor protein of the Cullin3 (Cul3)-dependent E3 ubiquitin ligase complex, forming the Keap1-Cul3-E3 complex with Cul3 (4). Under normal conditions, the Keap1-Cul3-E3 complex binds to Nrf2, sequestering it in the cytoplasm and promoting its ubiquitination and subsequent proteasomal degradation, thereby keeping Nrf2 at low levels. Upon oxidative stress, a conformational change occurs in the Keap1-Cul3-E3 complex, leading to the dissociation of Nrf2, which reduces its degradation and facilitates the nuclear translocation of Nrf2. In the nucleus, Nrf2 binds with small musculoaponeurotic fibrosarcoma protein (sMaf), forming the Nrf2-sMaf heterodimer, which then associates with the antioxidant response element (ARE) to initiate the transcription of antioxidant genes (4). These genes predominantly encode endogenous antioxidants, such as glutathione peroxidase (GPX), NAD(P)H:quinone oxidoreductase 1 (NQO1), superoxide dismutase (SOD) and various peroxidases such as CAT, all of which collaborate to preserve cellular redox balance and alleviate damage induced by oxidative stress. (See **Figure 1**)

## 3 Nrf2-mediated oxidative stress mechanisms in DR intervention

The onset and progression of DR are significantly influenced by oxidative stress, which refers to the disparity between oxidant production and the ability of the antioxidant system to neutralize them, which leads to redox dysregulation and the accumulation of excessive reactive oxygen species (ROS), such as hydroxyl radicals (·OH), triggering lipid peroxidation, protein oxidation and nucleic acid damage. Excessive ROS damages retinal blood vessels and surrounding tissues, inducing cell apoptosis, inflammatory responses, mitochondrial dysfunction and microvascular changes, ultimately leading to DR (5). In both DR patients and animal models, elevated levels of ROS in the retina and reduced levels of antioxidants such as SOD and reduced glutathione (GSH) have been observed (6), indicating that inhibiting ROS production and enhancing their elimination are critical strategies for DR treatment.

As a pivotal transcription factor in the oxidative stress defense system, Nrf2 mediates critical regulatory functions in DR pathogenesis. At the cellular level, high glucose treatment significantly elevates ROS levels in retinal pigment epithelial cells, while platycodin D (PLD) usage can reduce ROS levels and ameliorate DR by upregulating the Nrf2 pathway (7). Mesenchymal stem cells-derived exosomes (MSCs-EXOs) activate the PI3K/Akt pathway to govern Nrf2, leading to the upregulation of HO-1 and NQO1, thereby alleviating oxidative damage and cellular senescence in retinal pigment epithelial cells, as well as improving retinal structural damage (8). In animal models, diabetic mice exhibit reduced expression of Nrf2 in the eye, and topical application of the dipeptidyl peptidase-4 (DPP-4) inhibitor sitagliptin can restore Nrf2 and its downstream genes (e.g., CAT, SOD and GPX), with short-term (2 weeks) application improving early-stage DR in mice (9). Compared to anti-VEGF therapy, topical administration of DPP-4 inhibitors offers a non-invasive treatment approach that not only reduces the risk of complications but also alleviates the economic burden on patients, thus presenting a promising option for early DR prevention and intervention. Although currently the role of Nrf2 in antioxidant defense has been well established, the exact mechanisms underlying its regulation of ROS production pathways remain obscure, and further research should be conducted to delve into the differential mechanisms of oxidative stress and Nrf2 regulation across different retinal cell types. See **Table 1** for additional interventions of Nrf2 to alleviate oxidative stress. In conclusion, Nrf2 could ameliorate DR by countering oxidative stress through ROS reduction and antioxidant genes upregulation.

**Table 1:** Summary of drugs or treatments modulating Nrf2 pathways in DR intervention.

Drugs or Treatments	Experimental Cells or Animals	Effects Generated by Nrf2	Upstream Pathways	Effector Molecules	References
20(R)-ginsenoside Rg3	HRECs, mice	Anti-oxidative stress, anti-apoptosis, anti-ER stress	/	CAT, HO-1, SOD, BAX, Bcl-2, caspase-3, GRP78, p-PERK	Li, W., et al. (47)
APBOM	HUVECs, mice	Anti-oxidative stress, anti-angiogenesis	/	HO-1, NQO1, SOD, VEGF	Zhu, J., et al. (32)
Acteoside	ARPE-19, mice	Anti-oxidative stress	/	HO-1, NQO1	Yang, J., et al. (48)
Axitinib	HRECs	Anti-oxidative stress	/	ROS	Lazzara, F., et al. (49)
Sitagliptin	Mice	Anti-oxidative stress	/	CAT, GPX, GR, CuZnSOD, MnSOD	Ramos, H., et al. (9)
Astaxanthin	Rats	Anti-oxidative stress, anti-apoptosis, anti-inflammation	/	HO-1, γ-GCS, GPX, NQO1, BAX, Bcl-2, TNF-α, IL-1β, IL-6, MIC-1	Fang, J., et al. (13)
Astaxanthin	661W	Anti-oxidative stress, anti-apoptosis	PI3K/Akt/Nrf2	HO-1, NQO1, caspase-3	Lai, T.T., et al. (50)
Astragaloside-IV	ARPE-19	Anti-ferroptosis	miR-138-5p/Sirt1/Nrf2	GPX4, GCLC, GCLM	Tang, X., et al. (36)
CTRP9	ARPE-19	Anti-oxidative stress, anti-apoptosis	AMPK/Nrf2	HO-1, NQO1, SOD, BAX, Bcl-2, caspase-3	Cheng, Y., et al. (51)
CTRP3	HRPs	Anti-oxidative stress, anti-apoptosis	/	CAT, MnSOD, BAX, Bcl-2, caspase-3	Zeng, X., et al. (52)
CKIP-1	ARPE-19	Anti-oxidative stress, anti-apoptosis, anti-autophagy	p62/Keap1/Nrf2	SOD, Bad, BAX, Bcl-2, caspase-3, caspase-7, p62, Beclin-1, LC3	Zhao, X., et al. (46)
CGA	Microglia BV2, ARPE-19, mice	Anti-inflammation	/	TNF-α	Ouyang, H., et al. (28)
Corilagin	ARPE-19, mice	Anti-oxidative stress, anti-ferroptosis	/	HO-1, NQO1, GPX4, FTH-1, TFR, xCT	Shi, W., et al. (37)
Curcumin	Rats	Anti-oxidative stress, anti-apoptosis	/	HO-1, SOD	Xie, T., et al. (53)
Diosgenin	ARPEgen	Anti-oxidative stress, anti-apoptosis, anti-inflammation	AMPK/Nrf2	HO-1, GPX, SOD, BAX, Bcl-2, caspase-3, COX-2, TNF-α, IL-1β, IL-6	Hao, Y. and X. Gao (54)
DJ-1	RRPs	Anti-oxidative stress, anti-apoptosis	/	CAT, HO-1, NQO1, MnSOD, BAX, Bcl-2	Wang, W., et al. (55)
PHLPP1 siRNA	RGCs, rats	Anti-oxidative stress, anti-apoptosis	GSK-3β/Nrf2	ROS	Zhang, X., et al. (56)
Amygdalin	HRECs, rats	Anti-oxidative stress, anti-ferroptosis	/	CAT, GPX4, TFR1, ACSL4	Li, S., et al. (39)
Fractalkine	R28, BV2, rats	Anti-oxidative stress	fractalkine/CX3CR1/Nrf2	ROS	Jiang, M., et al. (57)
Flotillin− 1	ARPE-19, HUVECs, mice	Anti-ferroptosis	/	GPX4, xCT	Zhang, J., et al. (58)
miR-144 inhibitors	ARPE-19, mice	Anti-oxidative stress	/	GCLC, NQO1	Jadeja, R.N., et al. (59)
MLN4924	rRMECs, rats	Anti-oxidative stress, anti-inflammation	Inhibiting NEDD8-dependent neddylation of Cul3 to suppress ubiquitination and degradation of Nrf2	NQO1, caspase-1, IL-1β, NLRP3	Chen, Y., et al. (60)
miR-146a-5p	Rats	Anti-oxidative stress, anti-inflammation	/	HO-1, TNF-α	Rasoulinejad, S.A., et al. (61)
DHA	ARPE-19	Anti-oxidative stress	/	HO-1, NQO1	Bianchetti, G., et al. (62)
ALK7 siRNA	ARPEAN	Anti-oxidative stress, anti-apoptosis	/	CAT, HO-1, SOD, BAX, Bcl-2	Shi, Q., et al. (17)
GCN2 siRNA	ARPE-19	Anti-oxidative stress, anti-apoptosis	/	HO-1, SOD, BAX, Bcl-2, caspase-3	Zhang, X., et al. (18)
Liraglutide	Müller cells	Anti-oxidative stress, anti-apoptosis, anti-ER stress	p-Erk/Nrf2/Trx	GRP78, ATF6, p-PERK, IRE1	Ren, X., et al. (40)
lncRNA HOTAIR siRNA	HRECs	Anti-oxidative stress, anti-inflammation	/	GPX, HO-1, NQO1, SOD, caspase-1, IL-1β, GSDMD, NLRP3	You, H., et al. (26)
lncRNA MEG3	ARPE-19	Anti-apoptosis, anti-inflammation	miR-93/Nrf2	BAX, Bcl-2, caspase-3, IL-6, TNF-α	Luo, R., et al. (15)
lncRNA TPTEP1	HRVECs	Anti-oxidative stress	miR-489-3p/Nrf2	HO-1, NQO1	Wang, X., et al. (63)
lncRNA MALAT1 siRNA	HRECs, mice	Anti-oxidative stress	/	HO-1, NQO1, SOD2	Radhakrishnan, R. and R.A. Kowluru (64)
PHLPP2 shRNA	RGCs	Anti-oxidative stress, anti-apoptosis, anti-inflammation	Akt/GSK-3β/Nrf2	SOD, BAX, Bcl-2, IL-1β, IL-18, TNF-α	Liu, X., et al. (65)
STK25 shRNA	RGCs	Anti-oxidative stress, anti-apoptosis	Akt/GSK-3β/Nrf2	HO-1, NQO1, SOD, BAX, Bcl-2, caspase-3	Zhou, Z., et al. (66)
Lutein	ARPE-19	Anti-oxidative stress	PI3K/Akt, Erk1/2	CAT, HO-1, SOD2	Shivarudrappa, A.H. and G. Ponesakki (67)
Maresin-1	ARPE-19, mice	Anti-ferroptosis	/	ACSL4, GPX4, HO-1, PTGS2	Li, Y., et al. (38)
Maslinic acid	Rats	Anti-oxidative stress	/	SOD	Alsabaani, N.A., et al. (68)
MSCs-EXOs	ARPE-19, mice	Anti-oxidative stress	PI3K/Akt/Nrf2	HO-1, NQO1	Bai, L. and Y. Wang (8)
MSC-sEV	RPE, rats	Anti-oxidative stress, anti-apoptosis	PTEN/Akt/Nrf2	HO-1, GCLC, GCLM, GPX1, NQO1, BAX, Bcl-2, caspase-3	Sun, F., et al. (69)
miR-221 inhibitors	hRMECs	Anti-apoptosis	MiR-221/Sirt1/Nrf2	BAX, Bcl-2, caspase-3	Chen, B., et al. (16)
MSC-sEV	Müller cells, rRMECs, rats	Anti-oxidative stress, anti-inflammation	Inhibiting NEDD8-dependent neddylation of Cul3 to suppress ubiquitination and degradation of Nrf2	NQO1, caspase-1, IL-1β, IL-6, NLRP3	Chen, Y., et al. (24)
Platycodin D	ARPE-19	Anti-oxidative stress, anti-apoptosis	/	SOD, BAX, Bcl-2, caspase-3	Song, Y., et al. (7)
Polygonatum sibiricum polysaccharide	ARPE-19	Anti-oxidative stress, anti-apoptosis, anti-inflammation	/	HO-1, GPX, SOD, BAX, Bcl-2, caspase-3, IL-8, TNF-α	Wang, W., et al. (70)
Liraglutide	Neuro2a cells, mice	Anti-oxidative stress, anti-apoptosis, anti-ER stress	p-Erk/Nrf2/Trx	CHOP, GRP78, ATF4, p-PERK, IRE1	Liu, J., et al. (42)
NO_2_-OA	BAEC, MIO-M1	Anti-oxidative stress, anti-angiogenesis	/	HO-1, NQO1, VEGF	Vaglienti, M.V., et al. (30)
Sulforaphane	Rat Müller cells, rats	Anti-oxidative stress, anti-inflammation	/	CAT, HO-1, NQO1, SOD, IL-1β, IL-6, NLRP3, TNF-α	Li, S., et al. (25)
Bilobalide	Rats	Anti-oxidative stress	/	CAT, HO-1, SOD	Su, Q., et al. (71)
Resveratrol	Mice	Anti-oxidative stress, anti-apoptosis	/	HO-1, SOD, caspase-3	Yuan, D., et al. (72)
Resveratrol	Rats	Anti-ferroptosis	/	GPX4, PTGS2	Wang, Y., et al. (73)
Ad-SMP30	RGCs	Anti-oxidative stress, anti-apoptosis, anti-inflammation	Akt/GSK-3β/Nrf2	GPX, HO-1, NQO1, SOD, IL-1β, IL-6, TNF-α	Zhang, L., et al. (14)
Srxn1 cDNA	RGCs	Anti-oxidative stress, anti-apoptosis, anti-inflammation	Akt/GSK-3β/Nrf2	IL-1β, IL-6, TNF-α	Zhu, F., et al. (27)
lncRNA OGRU shRNA	HEK293T cells, Müller cells, rats	Anti-oxidative stress	miR-320/USP14/Nrf2	GCLC, GCLM, GPX, HO-1, NQO1, SOD	Fu, S., et al. (74)
Syringaresinol	RF/6A cells, mice	Anti-oxidative stress	/	HIF-1, HO-1, SOD2, VEGF	Liu, C., et al. (75)
Metoprolol	HRECs	Anti-oxidative stress	/	HO-1	Giurdanella, G., et al. (76)
PHLPP2 shRNA	Rats	Anti-oxidative stress, anti-apoptosis, anti-inflammation	Akt/GSK-3β/Nrf2	GPX, HO-1, NQO1, SOD, BAX, Bcl-2, IL-1β, IL-6, TNF-α	Chen, L., et al. (77)
MIND4N E	RGCs	Anti-oxidative stress	/	HO-1, NQO1	Chen, N., et al. (78)
TGF-β	RGCs	Anti-oxidative stress	/	HO-1	Chen, H.Y., et al. (79)
REDD1 knockdown	Müller cells, mice	Anti-oxidative stress	Akt/GSK-3β/Nrf2	GCLC, GCLM, HO-1, NQO1	Miller, W.P., et al. (80)
Tilianin	Rats	Anti-oxidative stress, anti-inflammation	/	CAT, GPX, HO-1, SOD, caspase-1, IL-1β, NLRP3, TXNIP	Zhang, Y., et al. (23)
Tricin	ARPE-19, rats	Anti-oxidative stress, anti-angiogenesis	Sestrin2/Nrf2	GPX, HO-1, SOD, VEGFR	Yang, X. and D. Li (31)
Brg1 cDNA	RGCs	Anti-oxidative stress, anti-apoptosis	Brg1/Nrf2	HO-1, caspase-3	Sun, W., et al. (81)
Urolithin A	HRECs	Anti-oxidative stress, anti-inflammation	/	HO-1, SOD, IL-1β, IL-6, TNF-α	Xu, Z., et al. (82)

## 4 Nrf2-mediated apoptotic mechanisms in DR intervention

Beyond oxidative stress, Nrf2 also regulates apoptosis mechanisms in DR. Cell apoptosis, the earliest identified pattern of programmed cell death, occurs via two essential mechanisms: the intrinsic and extrinsic pathways. BAX and Bak, as pro-apoptotic elements, increase mitochondrial permeability, which facilitates cytochrome c to be released into the cytoplasm, subsequently initiating a caspase cascade reaction, resulting in intrinsic apoptosis (10). Meanwhile, the binding of Fas ligand (FasL) and tumor necrosis factor-alpha (TNF-α) to their respective receptors activates a caspase cascade reaction, thereby inducing extrinsic apoptosis (11, 12).

Retinal cell apoptosis can occur in the early phases of DR, which often precedes other histopathological alterations (5). Studies on Nrf2-related apoptotic mechanisms in DR intervention have been conducted in recent years. In DR rat models, BAX expression is elevated, whereas Bcl-2 (as an anti-apoptotic factor) expression is reduced. Astaxanthin can trigger the Nrf2 signaling pathway to reverse these changes, thereby inhibiting apoptosis as well as thickening the retina and preserving retinal ganglion cell number, ultimately ameliorating DR (13). Senescence marker protein 30 (SMP30) is an aging-associated protein whose overexpression reduces high glucose-induced ganglion cell apoptosis via the Akt/GSK-3β/Nrf2 pathway, thereby exerting a protective effect on the retina (14). MiR-93 upregulation negatively regulates Nrf2 under hyperglycemic conditions, promoting apoptosis in retinal pigment epithelial cells, whereas long non-coding RNA (lncRNA) MEG3 enhances Nrf2 activity by complementarily interacting with miR-93, thereby reducing cell apoptosis (15). Additionally, miR-221 inhibits Nrf2 activation by suppressing Sirt1 expression, which results in elevated BAX and reduced Bcl-2 levels, thereby enhancing apoptosis in retinal microvascular endothelial cells (16). Given the differential gene expression among various retinal cell types, the overall impact of miRNA on retinal cell apoptosis requires further investigation. In a hyperglycemic environment, retinal pigment epithelial cells exhibit increased expression of ALK7 and GCN2 genes, while their knockdown suppresses cell apoptosis by activating the Nrf2/HO-1 pathway (17, 18). Future research should aim at delineating the interactions between Nrf2 and key apoptosis-related molecules, in order to provide more precise therapeutic targets for DR treatment. See **Table 1** for additional interventions of Nrf2 to inhibit cell apoptosis. In summary, Nrf2 exerts cytoprotective effects in DR by balancing pro- and anti-apoptotic factors to hinder cell apoptosis in retinal cells.

## 5 Nrf2-mediated inflammatory mechanisms in DR intervention

Apart from its anti-apoptotic effects, Nrf2 further demonstrates therapeutic potential by orchestrating an anti-inflammatory role. Both diabetic patients and animal models show the signs of chronic retinal inflammation, which could lead to vascular occlusion, neovascularization and macular edema, thereby accelerating the progression from NPDR to PDR (6, 19). In a hyperglycemic environment, excessive expression of pro-inflammatory molecules activates additional inflammatory cytokines and chemokines, leading to leukocyte aggregation, cell apoptosis and capillary leakage, thereby exacerbating tissue damage (20, 21). Current studies have linked various inflammatory cytokines, such as interleukin (IL)-1β, IL-6, IL-8, IL-12, IL-13, IL-17A, and TNF-α, to the severity of DR (6, 21, 22). Retinal cell dysfunction further amplifies inflammatory responses, which is a phenomenon that can be observed even in the early stages of DR (21).

Some progress has been made on Nrf2-related inflammatory mechanisms in DR intervention. In diabetic rats retinas, tilianin inhibits the elevation of thioredoxin interacting protein (TXNIP), NOD-like receptor protein 3 (NLRP3), caspase-1 and IL-1β levels via the Nrf2/TXNIP/NLRP3 inflammasome pathway, thereby mitigating retinal damage (23). MSC-derived small extracellular vesicles (MSC-sEV) can deliver miR-143-3p, which inhibits the neural precursor cell-expressed developmentally down-regulated 8 (NEDD8) neddylation of Cul3, thus preventing the proteasomal degradation of the Keap1-Cul3-E3-Nrf2 complex. This stabilizes Nrf2 and reduces the expression of NLRP3 and pro-inflammatory cytokines, exerting anti-inflammatory effects (24). The impact of other components within MSC-sEV on Nrf2 still requires further investigation. Sulforaphane, as an Nrf2 activator, could regulate Nrf2 signaling to suppress NLRP3 inflammasome formation, thus reducing levels of inflammatory cytokines to alleviate high-glucose-induced inflammatory damage in DR (25). In addition to causing the release of inflammatory cytokines, NLRP3 inflammasome could also trigger pyroptosis, a pattern of inflammation-driven cell death. LncRNA HOTAIR could inhibit Nrf2 under high glucose, resulting in NLRP3 inflammasome activation which subsequently induces pyroptosis, indicating that suppressing pyroptosis via Nrf2 activation could be an important therapeutic strategy for DR (26). In retinal ganglion cells exposed to high glucose, sulfiredoxin-1 (Srxn1) activates Nrf2 through the Akt/GSK-3β pathway, inhibiting the release of IL-1β, IL-6 and TNF-α (27). Additionally, chlorogenic acid (CGA) can reverse TNF-α-induced epithelial-mesenchymal transition (EMT), endothelial-mesenchymal transition (EndoMT) and blood-retinal barrier damage via Nrf2, with these effects being abolished when Nrf2 is inhibited (28). The molecular mechanisms linking Nrf2 with inflammatory factors, as well as the signaling pathways related to EMT and EndoMT, still require further exploration to determine the potential of Nrf2 as a therapeutic target in DR. See **Table 1** for additional interventions of Nrf2 to mitigate inflammation. In short, Nrf2-mediated downregulation of inflammatory cytokines highlights its anti-inflammatory potential, rendering Nrf2 an adequate target for alleviating chronic inflammation in DR.

## 6 Nrf2-mediated angiogenesis mechanisms in DR intervention

Beyond mitigating inflammation, Nrf2 activation exerts an essential function on retinal neovascularization, which is a key indicator of the transition from NPDR to PDR. VEGF is crucial in angiogenesis, facilitating endothelial cell migration and proliferation, enhancing leukocyte adhesion in the retina, and compromising the blood-retinal barrier. This disruption can result in endothelial cell damage, capillary hypoperfusion and the development of DME (22). Furthermore, VEGF increases vascular permeability by weakening intercellular tight junctions, thereby exacerbating blood-retinal barrier damage (29).

Previous studies have shown that nitro-oleic acid (NO_2_-OA) inhibits the expression of VEGF-A in retinal Müller cells via Nrf2, thereby attenuating angiogenesis induced by hypoxia and inflammation (30). Tricin activates the Sestrin2/Nrf2 pathway and downregulates the expression of VEGF receptor 2 (VEGFR2), which subsequently suppresses retinal morphological changes and neovascularization in diabetic rats. *In vitro* research has validated the anti-angiogenic effects of this pathway in retinal pigment epithelial cells (31). Research has also demonstrated that acidic polysaccharides from Buddleja officinalis (APBOM) significantly improve retinal pathological changes and neovascularization in diabetic mice by modulating the Nrf2 pathway (32). Future studies are required to further validate the role of these compounds in preventing retinal neovascularization via the Nrf2 pathway, in order to assess their clinical application potential. To conclude, pharmacological Nrf2 activation could attenuate retinal neovascularization, positioning Nrf2 agonists as promising adjuvants to conventional anti-VEGF therapies in DR management.

## 7 Nrf2-mediated ferroptosis mechanisms in DR intervention

Other than the above-mentioned cell death modes including apoptosis and pyroptosis, Nrf2 could also modulate ferroptosis, which is a unique type of cell death characterized by iron accumulation and lipid peroxidation (33). Iron ions induce massive ROS production via the Fenton reaction, initiating lipid peroxidation that drives cell ferroptosis ultimately (34). The system x_c_^-^ (composed of the subunits xCT and SLC3A2) and GPX4 are essential in regulating ferroptosis. The system x_c_^-^ imports cystine into cells, which is then utilized by γ-glutamate-cysteine ligase (γ-GCL), composed of the subunits GCLC and GCLM, to synthesize GSH. GPX4 catalyzes the elimination of lipid peroxides while converting GSH to its oxidized form (GSSG), thus safeguarding cells against ferroptosis (22).

Nrf2 is a key regulator of ferroptosis, which is involved in processes such as the synthesis and metabolism of GSH and GPX4, iron metabolism and lipid peroxidation (35). There has been abundant progress being made during recent years on Nrf2-related ferroptosis in DR intervention. High glucose treatment in retinal pigment epithelial cells elevates miR-138-5p expression, inhibiting the Sirt1/Nrf2 pathway and leading to ferroptosis. However, astragaloside-IV downregulates miR-138-5p, thereby activating the Sirt1/Nrf2 pathway, upregulating GPX4 and GCLC/GCLM and significantly alleviating ferroptosis (36). Corilagin can also inhibit ferroptosis by activating Nrf2, reducing cell tight junction damage. After silencing Nrf2, xCT and GPX4 levels decrease, while transferrin receptor (TFR) and ferritin heavy chain (FTH) levels increase (37). Furthermore, in both *in vivo* and *in vitro* experiments, Maresin-1 significantly inhibits high glucose-induced ferroptosis by activating the Nrf2/HO-1/GPX4 pathway, which is manifested by increased GPX4 levels and downregulation of the ferroptosis-promoting factor acyl-CoA synthetase long-chain family member 4 (ACSL4) (38). By activating Nrf2 in retinal endothelial cells, amygdalin suppresses ferroptosis and delays the progression of DR in rats (39). Future research should further explore the potential of targeting Nrf2 to regulate ferroptosis in various retinal cell types and investigate the interactions between Nrf2 and other ferroptosis-associated pathways to expand therapeutic strategies for DR. See **Table 1** for additional interventions of Nrf2 to reduce ferroptosis. Overall, Nrf2 safeguards retinal cells against ferroptosis by modulating GPX4-mediated elimination of lipid peroxidation resulted from iron accumulation, designating inhibition of ferroptosis via Nrf2 as a possible means of improving DR.

## 8 Nrf2-mediated endoplasmic reticulum stress and autophagy in DR intervention

The multifaceted protective role of Nrf2 could also extend to the regulation of endoplasmic reticulum (ER) stress and autophagy, further expanding its therapeutic landscape in DR. Misfolded or unfolded proteins are typically degraded via the ubiquitin-proteasome system or autophagy to maintain protein homeostasis. However, when these proteins cannot be cleared promptly, ER stress can be subsequently induced by three transmembrane proteins in the ER: inositol-requiring enzyme 1 (IRE1), protein kinase R-like endoplasmic reticulum kinase (PERK), and activating transcription factor 6 (ATF6) (40). ER stress in the pathophysiology of DR is primarily associated with blood-retinal barrier disruption, retinal neovascularization and neuronal injury (41). Liraglutide has been shown to inhibit ER stress, positioning it as a potential candidate for DR prevention and therapies. Under high-glucose conditions in Neuro2a cells treated with liraglutide, the activation of the p-Erk/Nrf2/Trx pathway decreases ER stress proteins such as IRE1 and PERK (42). Similarly, in high-glucose-treated Müller cells, liraglutide significantly inhibits ER stress through activation of this pathway (40). See **Table 1** for additional interventions of Nrf2 to reduce ER stress. In brief, ER stress inhibition by Nrf2 activation may emerge as a novel means of combating DR.

Autophagy is a cellular protective mechanism, through which the lysosome degrades cytoplasm, proteins and damaged organelles as well as metabolic waste and aging by-products, and subsequently the resulting products can be recycled in cells. This process is vital for cellular homeostasis and significantly influences the pathogenesis and progression of DR (43–45). The selective autophagy receptor p62 can competitively bind to Keap1, thereby promoting Nrf2 activation. It has been demonstrated that high-glucose-treated human retinal pigment epithelial cells exhibit reduced CKIP-1 levels, elevated autophagy markers, and diminished p62 accumulation, indicating autophagy activation, whereas CKIP-1 overexpression activates the p62/Keap1/Nrf2 axis and significantly suppresses cell autophagy (46). Thus, modulating autophagy through the p62/Keap1/Nrf2 pathway offers a promising approach to improving the progression of DR.

## 9 Conclusions and future perspectives

DR has become one of the leading causes of vision loss in diabetic patients, with its pathogenesis involving processes such as oxidative stress and chronic inflammation. Nrf2 alleviates oxidative damage by activating antioxidant genes and reducing intracellular ROS accumulation. Nrf2 also inhibits inflammatory responses, cell apoptosis, angiogenesis and ferroptosis, thereby improving retinal damage. It is noteworthy that these mechanisms regulated by Nrf2 are interconnected and exhibit mutual interactions, implying that intervention of Nrf2 may yield multiple ameliorative effects on DR rather than exerting a single mechanism separately. **Table 1** summarizes the effects of pharmacological interventions targeting Nrf2 in improving DR, along with its upstream pathways and downstream molecules. **Figure 1** illustrates the mechanism of the Keap1-Nrf2-ARE pathway, along with interventions targeting its upstream pathways and the effects on Nrf2.

Given the limited efficacy of current treatment options (such as anti-VEGF therapy) in certain patients, Nrf2, as a pleiotropic target, holds potential as a complementary therapeutic strategy alongside conventional treatments, rather than as a replacement. Notably, most of the research cited in this review is based on preclinical studies, and currently no clinical trials have been conducted on the use of Nrf2 modulators for the treatment of DR, which is primarily due to the challenges associated with translating Nrf2 activators to clinical applications. For example, the toxicity and adverse effects of Nrf2 activators have not been fully elucidated. Further studies focusing on Nrf2-related animal models are warranted to better characterize the toxicological profiles, pharmacokinetics and pharmacodynamics of these compounds. In addition, the development of Nrf2 activator analogs with reduced toxicity and side effects would be crucial for advancing Nrf2-mediated therapies for DR. To translate Nrf2-based therapies to clinical application, more clinical trials should be conducted. Among the strategies of intervening Nrf2, some safe nutraceuticals such as lutein and curcumin stand out to be more promising for early clinical trials compared to therapies based on gene regulation, owing to their relative safety and easy accessibility from daily diet. To date, no drugs specifically targeting Nrf2 have been developed. Future studies should investigate the regulatory roles of Nrf2 throughout various stages of DR, focusing on its interactions with cell apoptosis, autophagy, ferroptosis and angiogenesis, to further elucidate the signaling pathways involved, thereby offering innovative therapeutic strategies for DR prevention and treatment.

## Glossary

ACSL4: Acyl-CoA synthetase long-chain family member 4
APAF-1: Apoptotic protease activating factor-1
ARE: Antioxidant response element
ATF: Activating transcription factor
BAEC: Bovine aortic endothelial cell
CAT: Catalase
CGA: Chlorogenic acid
CKIP-1: Casein kinase 2-interacting protein-1
COX-2: Cyclooxygenase-2
CTRP: C1q/TNF-related protein
DME: Diabetic macular edema
DPP-4: Dipeptidyl peptidase-4
DR: Diabetic retinopathy
EMT: Epithelial-mesenchymal transition
EndoMT: Endothelial-mesenchymal transition
FasL: Fas ligand
FTH: Ferritin heavy chain
γ-GCL: γ-Glutamate-cysteine ligase
GCLC: Glutamate-cysteine ligase catalytic subunit
GCLM: Glutamate-cysteine ligase modifier subunit
GPX: Glutathione peroxidase
GR: Glutathione reductase
GSDMD: Gasdermin D
GSH: Reduced glutathione
GSSG: Oxidized glutathione
HO-1: Heme oxygenase-1
HREC: Human retinal endothelial cell
hRMEC: Human retinal microvascular endothelial cell
HRPs: Human retinal pericytes
HRVECs: Human retinal vascular endothelial cell
HUVECs: Human umbilical vein endothelial cells
IRE1: Inositol-requiring enzyme 1
Keap1: Kelch-like ECH-associated protein 1
lncRNA: Long non-coding RNA
MIC-1: Macrophage inhibitory cytokine-1
miRNA: microRNA
MSC-sEV: Mesenchymal stem cells-derived small extracellular vesicles
MSCs-EXOs: Mesenchymal stem cells-derived exosomes
NO_2_-OA: Nitro-oleic acid
NPDR: Non-proliferative DR
NQO1: NAD(P)H:quinone oxidoreductase 1
Nrf2: Nuclear factor E2-related factor 2
PDR: Proliferative DR
PERK: Protein kinase R-like endoplasmic reticulum kinase
PLD: Platycodin D
PTGS2: Prostaglandin-endoperoxide synthase 2
PUFAs: Polyunsaturated fatty acids
RGCs: Retinal ganglion cells
ROS: Reactive oxygen species
RPE: Retinal pigment epithelial cells
rRMEC: Rat retinal microvascular endothelial cell
RRPs: Rat retinal pericytes
shRNA: Short hairpin RNA
siRNA: Small interfering RNA
SMP30: Senescence marker protein 30
SOD: Superoxide dismutase
Srxn1: Sulfiredoxin-1
STK25: Serine/threonine protein kinase 25
TFR: Transferrin receptor
TNF-α: Tumor necrosis factor-alpha
TNFR: TNF receptor
TXNIP: Thioredoxin interacting protein
VEGF: Vascular endothelial growth factor.

## References

[1] Yau JW Rogers SL Kawasaki R Lamoureux EL Kowalski JW Bek T. Global prevalence and major risk factors of diabetic retinopathy. Diabetes Care. (2012) 35:556–64. doi: 10.2337/dc11-1909 PMC3322721 22301125

[2] Cheung N Mitchell P Wong TY. Diabetic retinopathy. Lancet. (2010) 376:124–36. doi: 10.1016/s0140-6736(09)62124-3 20580421

[3] Antonetti DA Silva PS Stitt AW. Current understanding of the molecular and cellular pathology of diabetic retinopathy. Nat Rev Endocrinol. (2021) 17:195–206. doi: 10.1038/s41574-020-00451-4 33469209 PMC9053333

[4] Chen J Wang Q Li R Li Z Jiang Q Yan F. The role of Keap1-Nrf2 signaling pathway during the progress and therapy of diabetic retinopathy. Life Sci. (2024) 338:122386. doi: 10.1016/j.lfs.2023.122386 38159594

[5] Kang Q Yang C. Oxidative stress and diabetic retinopathy: molecular mechanisms, pathogenetic role and therapeutic implications. Redox Biol. (2020) 37:101799. doi: 10.1016/j.redox.2020.101799 33248932 PMC7767789

[6] Li H Liu X Zhong H Fang J Li X Shi R. Research progress on the pathogenesis of diabetic retinopathy. BMC Ophthalmol. (2023) 23:372. doi: 10.1186/s12886-023-03118-6 37697295 PMC10494348

[7] Song Y Lv P Yu J. Platycodin D inhibits diabetic retinopathy via suppressing TLR4/Myd88/NF-Κb signaling pathway and activating Nrf2/HO-1 signaling pathway. Chem Biol Drug Des. (2024) 103:e14419. doi: 10.1111/cbdd.14419 38230792

[8] Bai L Wang Y. Mesenchymal stem cells-derived exosomes alleviate senescence of retinal pigment epithelial cells by activating PI3K/AKT-Nrf2 signaling pathway in early diabetic retinopathy. Exp Cell Res. (2024) 441:114170. doi: 10.1016/j.yexcr.2024.114170 39019426

[9] Ramos H Bogdanov P Huerta J Deàs-Just A Hernández C Simó R. Antioxidant effects of DPP-4 inhibitors in early stages of experimental diabetic retinopathy. Antioxidants (Basel). (2022) 11:1418. doi: 10.3390/antiox11071418 35883908 PMC9312245

[10] Bertheloot D Latz E Franklin BS. Necroptosis, pyroptosis and apoptosis: an intricate game of cell death. Cell Mol Immunol. (2021) 18:1106–21. doi: 10.1038/s41423-020-00630-3 PMC8008022 33785842

[11] Tang D Kang R Berghe TV Vandenabeele P Kroemer G. The molecular machinery of regulated cell death. Cell Res. (2019) 29:347–64. doi: 10.1038/s41422-019-0164-5 PMC6796845 30948788

[12] Jaeschke H Ramachandran A Chao X Ding WX. Emerging and established modes of cell death during acetaminophen-induced liver injury. Arch Toxicol. (2019) 93:3491–502. doi: 10.1007/s00204-019-02597-1 PMC6891214 31641808

[13] Fang J Bai W Yang L. Astaxanthin inhibits oxidative stress and apoptosis in diabetic retinopathy. Acta Histochem. (2023) 125:152069. doi: 10.1016/j.acthis.2023.152069 37343496

[14] Zhang L Zhu T He F Li X. Senescence marker protein 30 (SMP30) protects against high glucose-induced apoptosis, oxidative stress and inflammatory response in retinal ganglion cells by enhancing Nrf2 activation via regulation of Akt/GSK-3β Pathway. Int Immunopharmacol. (2021) 101:108238. doi: 10.1016/j.intimp.2021.108238 34688152

[15] Luo R Jin H Li L Hu YX Xiao F. Long noncoding RNA MEG3 inhibits apoptosis of retinal pigment epithelium cells induced by high glucose via the miR-93/Nrf2 axis. Am J Pathol. (2020) 190:1813–22. doi: 10.1016/j.ajpath.2020.05.008 32473920

[16] Chen B Wu L Cao T Zheng HM He T. MiR-221/Sirt1/Nrf2 signal axis regulates high glucose induced apoptosis in human retinal microvascular endothelial cells. BMC Ophthalmol. (2020) 20:300. doi: 10.1186/s12886-020-01559-x 32698791 PMC7374880

[17] Shi Q Dong X Zhang M Cheng Y Pei C. Knockdown of ALK7 inhibits high glucose-induced oxidative stress and apoptosis in retinal pigment epithelial cells. Clin Exp Pharmacol Physiol. (2020) 47:313–21. doi: 10.1111/1440-1681.13189 31608496

[18] Zhang X He N Xing Y Lu Y. Knockdown of GCN2 inhibits high glucose-induced oxidative stress and apoptosis in retinal pigment epithelial cells. Clin Exp Pharmacol Physiol. (2020) 47:591–8. doi: 10.1111/1440-1681.13233 31868938

[19] Soto I Krebs MP Reagan AM Howell GR. Vascular inflammation risk factors in retinal disease. Annu Rev Vis Sci. (2019) 5:99–122. doi: 10.1146/annurev-vision-091517-034416 31226014

[20] Taurone S Ralli M Nebbioso M Greco A Artico M Attanasio G. The role of inflammation in diabetic retinopathy: A review. Eur Rev Med Pharmacol Sci. (2020) 24:10319–29. doi: 10.26355/eurrev_202010_23379 33155187

[21] Tang L Xu GT Zhang JF. Inflammation in diabetic retinopathy: possible roles in pathogenesis and potential implications for therapy. Neural Regener Res. (2023) 18:976–82. doi: 10.4103/1673-5374.355743 PMC9827774 36254977

[22] He W Chang L Li X Mei Y. Research progress on the mechanism of ferroptosis and its role in diabetic retinopathy. Front Endocrinol (Lausanne). (2023) 14:1155296. doi: 10.3389/fendo.2023.1155296 37334304 PMC10268817

[23] Zhang Y Gao Z Gao X Yuan Z Ma T Li G. Tilianin protects diabetic retina through the modulation of Nrf2/TXNIP/NLRP3 inflammasome pathways. J Environ Pathol Toxicol Oncol. (2020) 39:89–99. doi: 10.1615/JEnvironPatholToxicolOncol.2020032544 32479015

[24] Chen Y Tong J Liu C He C Xiang J Yao G. MSC-derived small extracellular vesicles mitigate diabetic retinopathy by stabilizing Nrf2 through miR-143-3p-mediated inhibition of neddylation. Free Radic Biol Med. (2024) 219:76–87. doi: 10.1016/j.freeradbiomed.2024.04.216 38604315

[25] Li S Yang H Chen X. Protective effects of sulforaphane on diabetic retinopathy: activation of the Nrf2 pathway and inhibition of NLRP3 inflammasome formation. Exp Anim. (2019) 68:221–31. doi: 10.1538/expanim.18-0146 PMC6511524 30606939

[26] You H Li H Gou W. lncRNA hotair promotes ROS generation and NLRP3 inflammasome activation by inhibiting Nrf2 in diabetic retinopathy. Med (Baltimore). (2023) 102:e35155. doi: 10.1097/md.0000000000035155 PMC10508377 37713847

[27] Zhu F Shao J Tian Y Xu Z. Sulfiredoxin-1 protects retinal ganglion cells from high glucose-induced oxidative stress and inflammatory injury by potentiating Nrf2 signaling via the Akt/GSK-3β Pathway. Int Immunopharmacol. (2021) 101:108221. doi: 10.1016/j.intimp.2021.108221 34653733

[28] Ouyang H Du A Zhou L Zhang T Lu B Wang Z. Chlorogenic acid improves diabetic retinopathy by alleviating blood-retinal-barrier dysfunction via inducing Nrf2 activation. Phytother Res. (2022) 36:1386–401. doi: 10.1002/ptr.7401 35133045

[29] Murakami T Frey T Lin C Antonetti DA. Protein kinase Cβ Phosphorylates occludin regulating tight junction trafficking in vascular endothelial growth factor-induced permeability *in vivo* . Diabetes. (2012) 61:1573–83. doi: 10.2337/db11-1367 PMC3357276 22438576

[30] Vaglienti MV Subirada PV Joray MB Bonacci G Sánchez MC. Protective effect of NO(2)-OA on oxidative stress, gliosis, and pro-angiogenic response in müller glial cells. Cells. (2023) 12:494. doi: 10.3390/cells12030494 36766836 PMC9914399

[31] Yang X Li D. Tricin attenuates diabetic retinopathy by inhibiting oxidative stress and angiogenesis through regulating sestrin2/nrf2 signaling. Hum Exp Toxicol. (2023) 42:9603271231171642. doi: 10.1177/09603271231171642 37077025

[32] Zhu J Sun H Kang X Zhu H Yan X. Acidic polysaccharides from buddleja officinalis inhibit angiogenesis via the Nrf2/ARE pathway to attenuate diabetic retinopathy. Food Funct. (2022) 13:9021–31. doi: 10.1039/d2fo01075e 35942925

[33] Dixon SJ Lemberg KM Lamprecht MR Skouta R Zaitsev EM Gleason CE. Ferroptosis: an iron-dependent form of nonapoptotic cell death. Cell. (2012) 149:1060–72. doi: 10.1016/j.cell.2012.03.042 PMC3367386 22632970

[34] Stockwell BR. Ferroptosis turns 10: emerging mechanisms, physiological functions, and therapeutic applications. Cell. (2022) 185:2401–21. doi: 10.1016/j.cell.2022.06.003 PMC9273022 35803244

[35] Han H Zhang G Zhang X Zhao Q. Nrf2-mediated ferroptosis inhibition: A novel approach for managing inflammatory diseases. Inflammopharmacology. (2024) 32:2961–86. doi: 10.1007/s10787-024-01519-7 39126567

[36] Tang X Li X Zhang D Han W. Astragaloside-iv alleviates high glucose-induced ferroptosis in retinal pigment epithelial cells by disrupting the expression of miR-138-5p/Sirt1/Nrf2. Bioengineered. (2022) 13:8240–54. doi: 10.1080/21655979.2022.2049471 PMC9162003 35302431

[37] Shi W Dong Y Liu S Li F Zhu C. Corilagin alleviates ferroptosis in diabetic retinopathy by activating the Nrf2 signaling pathway. BioMed Pharmacother. (2024) 179:117409. doi: 10.1016/j.biopha.2024.117409 39243434

[38] Li Y Liu J Ma X Bai X. Maresin-1 inhibits high glucose induced ferroptosis in ARPE-19 cells by activating the Nrf2/HO-1/GPX4 pathway. BMC Ophthalmol. (2023) 23:368. doi: 10.1186/s12886-023-03115-9 37674121 PMC10481498

[39] Li S Lu S Wang L Liu S Zhang L Du J. Effects of amygdalin on ferroptosis and oxidative stress in diabetic retinopathy progression via the Nrf2/are signaling pathway. Exp Eye Res. (2023) 234:109569. doi: 10.1016/j.exer.2023.109569 37422064

[40] Ren X Sun L Wei L Liu J Zhu J Yu Q. Liraglutide up-regulation thioredoxin attenuated müller cells apoptosis in high glucose by regulating oxidative stress and endoplasmic reticulum stress. Curr Eye Res. (2020) 45:1283–91. doi: 10.1080/02713683.2020.1737137 32180468

[41] Chen X Shi C He M Xiong S Xia X. Endoplasmic reticulum stress: molecular mechanism and therapeutic targets. Signal Transduct Target Ther. (2023) 8:352. doi: 10.1038/s41392-023-01570-w 37709773 PMC10502142

[42] Liu J Wei L Wang Z Song S Lin Z Zhu J. Protective effect of liraglutide on diabetic retinal neurodegeneration via inhibiting oxidative stress and endoplasmic reticulum stress. Neurochem Int. (2020) 133:104624. doi: 10.1016/j.neuint.2019.104624 31794832

[43] Fu D Yu JY Yang S Wu M Hammad SM Connell AR. Survival or death: A dual role for autophagy in stress-induced pericyte loss in diabetic retinopathy. Diabetologia. (2016) 59:2251–61. doi: 10.1007/s00125-016-4058-5 PMC5016562 27475954

[44] Yang X Huang Z Xu M Chen Y Cao M Yi G. Autophagy in the retinal neurovascular unit: new perspectives into diabetic retinopathy. J Diabetes. (2023) 15:382–96. doi: 10.1111/1753-0407.13373 PMC10172025 36864557

[45] Rosa MD Distefano G Gagliano C Rusciano D Malaguarnera L. Autophagy in diabetic retinopathy. Curr Neuropharmacol. (2016) 14:810–25. doi: 10.2174/1570159x14666160321122900 PMC5333581 26997506

[46] Zhao X Wang J Li P Tang L Bai Y. Casein kinase 2-interacting protein-1 alleviates high glucose-reduced autophagy, oxidative stress, and apoptosis in retinal pigment epithelial cells via activating the P62/KEAP1/NRF2 signaling pathway. J Ophthalmol. (2021) 2021:6694050. doi: 10.1155/2021/6694050 33628480 PMC7892229

[47] Li WL Li K Chang WG Shi H Zhang WX Wang Z. 20(R)-ginsenoside Rg3 alleviates diabetic retinal injury in T2DM mice by attenuating ros-mediated er stress through the activation of the Nrf2/HO-1 axis. Phytomedicine. (2024) 135:156202. doi: 10.1016/j.phymed.2024.156202 39579577

[48] Yang J Hua Z Zheng Z Ma X Zhu L Li Y. Acteoside inhibits high glucose-induced oxidative stress injury in RPE cells and the outer retina through the KEAP1/Nrf2/are pathway. Exp Eye Res. (2023) 232:109496. doi: 10.1016/j.exer.2023.109496 37268044

[49] Lazzara F Conti F Sasmal PK Alikunju S Rossi S Drago F. Anti-angiogenic and antioxidant effects of axitinib in human retinal endothelial cells: implications in diabetic retinopathy. Front Pharmacol. (2024) 15:1415846. doi: 10.3389/fphar.2024.1415846 38953109 PMC11215076

[50] Lai TT Yang CM Yang CH. Astaxanthin protects retinal photoreceptor cells against high glucose-induced oxidative stress by induction of antioxidant enzymes via the PI3k/Akt/Nrf2 pathway. Antioxidants (Basel). (2020) 9:729. doi: 10.3390/antiox9080729 32785112 PMC7465141

[51] Cheng Y Qi Y Liu S Di R Shi Q Li J. C1q/TNF-related protein 9 inhibits high glucose-induced oxidative stress and apoptosis in retinal pigment epithelial cells through the activation of ampk/nrf2 signaling pathway. Cell Transplant. (2020) 29:963689720962052. doi: 10.1177/0963689720962052 33040597 PMC7784607

[52] Zeng X Peng Y Wang Y Kang K. C1q/tumor necrosis factor-related protein-3 (CTRP3) activated by forkhead box o4 (FOXO4) down-regulation protects retinal pericytes against high glucose-induced oxidative damage through nuclear factor erythroid 2-related factor 2 (Nrf2)/Nuclear factor-kappab (NF-Κb) signaling. Bioengineered. (2022) 13:6080–91. doi: 10.1080/21655979.2022.2031413 PMC8974204 35196182

[53] Xie T Chen X Chen W Huang S Peng X Tian L. Curcumin is a potential adjuvant to alleviates diabetic retinal injury via reducing oxidative stress and maintaining Nrf2 pathway homeostasis. Front Pharmacol. (2021) 12:796565. doi: 10.3389/fphar.2021.796565 34955862 PMC8702852

[54] Hao Y Gao X. Diosgenin protects retinal pigment epithelial cells from inflammatory damage and oxidative stress induced by high glucose by activating AMPK/Nrf2/HO-1 pathway. Immun Inflammation Dis. (2022) 10:e698. doi: 10.1002/iid3.698 PMC9667204 36444632

[55] Wang W Zhao H Chen B. Dj-1 Protects Retinal Pericytes against High Glucose-Induced Oxidative Stress through the Nrf2 Signaling Pathway. Sci Rep. (2020) 10:2477. doi: 10.1038/s41598-020-59408-2 32051471 PMC7016111

[56] Zhang X Lu Y He N Wang F. Downregulation of phlpp1 ameliorates high glucose-evoked injury in retinal ganglion cells by attenuating apoptosis and oxidative stress through enhancement of Nrf2 activation. Exp Cell Res. (2020) 397:112344. doi: 10.1016/j.yexcr.2020.112344 33164862

[57] Jiang M Xie H Zhang C Wang T Tian H Lu L. Enhancing fractalkine/CX3CR1 signalling pathway can reduce neuroinflammation by attenuating microglia activation in experimental diabetic retinopathy. J Cell Mol Med. (2022) 26:1229–44. doi: 10.1111/jcmm.17179 PMC8831940 35023309

[58] Zhang J Chang K Shangguan Y Luo R Bi Y Yu Z. Flotillin- 1 ameliorates experimental diabetic retinopathy by inhibiting ferroptosis in blood-retinal barrier. J Mol Med (Berl). (2025). doi: 10.1007/s00109-025-02544-x 40198383

[59] Jadeja RN Jones MA Abdelrahman AA Powell FL Thounaojam MC Gutsaeva D. Inhibiting microrna-144 potentiates Nrf2-dependent antioxidant signaling in RPE and protects against oxidative stress-induced outer retinal degeneration. Redox Biol. (2020) 28:101336. doi: 10.1016/j.redox.2019.101336 31590045 PMC6812120

[60] Chen Y Liu C Tong J He C Ling X Xiang J. Inhibition of cullin3 neddylation alleviates diabetic retinopathy by activating Nrf2 signaling to combat ROS-induced oxidative stress and inflammation. Inflammation. (2025). doi: 10.1007/s10753-025-02259-8 PMC12596376 40021543

[61] Rasoulinejad SA Akbari A Nasiri K. Interaction of miR-146a-5p with oxidative stress and inflammation in complications of type 2 diabetes mellitus in male rats: anti-oxidant and anti-inflammatory protection strategies in type 2 diabetic retinopathy. Iran J Basic Med Sci. (2021) 24:1078–86. doi: 10.22038/ijbms.2021.56958.12706 PMC8591764 34804425

[62] Bianchetti G Clementi ME Sampaolese B Serantoni C Abeltino A De Spirito M. Investigation of DHA-induced regulation of redox homeostasis in retinal pigment epithelium cells through the combination of metabolic imaging and molecular biology. Antioxidants (Basel). (2022) 11:1072. doi: 10.3390/antiox11061072 35739970 PMC9219962

[63] Wang X Zhou X Wang F Zhang N Zhang Y Ao Z. Long noncoding RNA TPTEP1 suppresses diabetic retinopathy by reducing oxidative stress and targeting the mir-489-3p/NRF2 axis. Acta Biochim Pol. (2023) 70:45–50. doi: 10.18388/abp.2020_6034 36795778

[64] Radhakrishnan R Kowluru RA. Long noncoding RNA MALAT1 and regulation of the antioxidant defense system in diabetic retinopathy. Diabetes. (2021) 70:227–39. doi: 10.2337/db20-0375 PMC7881848 33051272

[65] Liu X Liu Y Chen L Zhang Z Cui L Wei T. Loss of pleckstrin homology domain and leucine-rich repeat protein phosphatase 2 has protective effects on high glucose-injured retinal ganglion cells via the effect on the Akt-GSK-3β-Nrf2 pathway. Inflammation Res. (2023) 72:373–85. doi: 10.1007/s00011-022-01680-1 36562794

[66] Zhou Z Li H Bai S Xu Z Jiao Y. Loss of serine/threonine protein kinase 25 in retinal ganglion cells ameliorates high glucose-elicited damage through regulation of the Akt-GSK-3β-Nrf2 pathway. Biochem Biophys Res Commun. (2022) 600:87–93. doi: 10.1016/j.bbrc.2022.02.044 35217361

[67] Shivarudrappa AH Ponesakki G. Lutein reverses hyperglycemia-mediated blockage of Nrf2 translocation by modulating the activation of intracellular protein kinases in retinal pigment epithelial (ARPE-19) cells. J Cell Commun Signal. (2020) 14:207–21. doi: 10.1007/s12079-019-00539-1 PMC7272517 31820335

[68] Alsabaani NA Osman OM Dallak MA Morsy MD Al-Dhibi HA. Maslinic acid protects against streptozotocin-induced diabetic retinopathy by activating Nrf2 and suppressing NF-Κb. J Ophthalmol. (2022) 2022:3044202. doi: 10.1155/2022/3044202 35265366 PMC8901311

[69] Sun F Sun Y Zhu J Wang X Ji C Zhang J. Mesenchymal stem cells-derived small extracellular vesicles alleviate diabetic retinopathy by delivering NEDD4. Stem Cell Res Ther. (2022) 13:293. doi: 10.1186/s13287-022-02983-0 35841055 PMC9284871

[70] Wang W Li S Song M. Polygonatum sibiricum polysaccharide inhibits high glucose-induced oxidative stress, inflammatory response, and apoptosis in RPE cells. J Recept Signal Transduct Res. (2022) 42:189–96. doi: 10.1080/10799893.2021.1883061 33554697

[71] Su Q Dong J Zhang D Yang L Roy R. Protective effects of the bilobalide on retinal oxidative stress and inflammation in streptozotocin-induced diabetic rats. Appl Biochem Biotechnol. (2022) 194:6407–22. doi: 10.1007/s12010-022-04012-5 35932369

[72] Yuan D Xu Y Xue L Zhang W Gu L Liu Q. Resveratrol protects against diabetic retinal ganglion cell damage by activating the Nrf2 signaling pathway. Heliyon. (2024) 10:e30786. doi: 10.1016/j.heliyon.2024.e30786 38774075 PMC11107105

[73] Wang Y Song SY Song Y Wang Y Wan ZW Sun P. Resveratrol protects müller cells against ferroptosis in the early stage of diabetic retinopathy by regulating the Nrf2/GPX4/PTGS2 pathway. Mol Neurobiol. (2025) 62:3412–27. doi: 10.1007/s12035-024-04496-8 39292340

[74] Fu S Zheng Y Sun Y Lai M Qiu J Gui F. Suppressing long noncoding RNA OGRU ameliorates diabetic retinopathy by inhibition of oxidative stress and inflammation via miR-320/USP14 axis. Free Radic Biol Med. (2021) 169:361–81. doi: 10.1016/j.freeradbiomed.2021.03.016 33762162

[75] Liu C Cheng T Wang Y Li G Wang Y Tian W. Syringaresinol alleviates early diabetic retinopathy by downregulating HIF-1α/VEGF via activating Nrf2 antioxidant pathway. Mol Nutr Food Res. (2024) 68:e2200771. doi: 10.1002/mnfr.202200771 38356045

[76] Giurdanella G Longo A Distefano A Olivieri M Cristaldi M Cosentino A. The anti-inflammatory effect of the Β1-adrenergic receptor antagonist metoprolol on high glucose treated human microvascular retinal endothelial cells. Cells. (2021) 11:51. doi: 10.3390/cells11010051 35011613 PMC8750370

[77] Chen L Qi E Liu X Cui L Fan X Wei T. The lack of homology domain and leucine rich repeat protein phosphatase 2 ameliorates visual impairment in rats with diabetic retinopathy through regulation of the AKT-GSK-3β-Nrf2 signal cascade. Toxicol Appl Pharmacol. (2024) 482:116766. doi: 10.1016/j.taap.2023.116766 37995808

[78] Chen N Li Y Huang N Yao J Luo WF Jiang Q. The Nrf2 activator MIND4–17 protects retinal ganglion cells from high glucose-induced oxidative injury. J Cell Physiol. (2020) 235:7204–13. doi: 10.1002/jcp.29619 32020639

[79] Chen HY Ho YJ Chou HC Liao EC Tsai YT Wei YS. The role of transforming growth factor-beta in retinal ganglion cells with hyperglycemia and oxidative stress. Int J Mol Sci. (2020) 21:6482. doi: 10.3390/ijms21186482 32899874 PMC7554964

[80] Miller WP Sunilkumar S Giordano JF Toro AL Barber AJ Dennis MD. The stress response protein REDD1 promotes diabetes-induced oxidative stress in the retina by Keap1-independent Nrf2 degradation. J Biol Chem. (2020) 295:7350–61. doi: 10.1074/jbc.RA120.013093 PMC7247303 32295843

[81] Sun W Yu J Kang Q. Upregulation of heme oxygenase-1 by brahma-related gene 1 through Nrf2 signaling confers protective effect against high glucose-induced oxidative damage of retinal ganglion cells. Eur J Pharmacol. (2020) 875:173038. doi: 10.1016/j.ejphar.2020.173038 32105681

[82] Xu Z Li S Li K Wang X Li X An M. Urolithin a ameliorates diabetic retinopathy via activation of the Nrf2/HO-1 pathway. Endocr J. (2022) 69:971–82. doi: 10.1507/endocrj.EJ21-0490 35321989

